# Tree size and its relationship with flowering phenology and reproductive output in Wild Nutmeg trees

**DOI:** 10.1002/ece3.742

**Published:** 2013-08-29

**Authors:** Mauricio Fernández Otárola, Marlies Sazima, Vera N Solferini

**Affiliations:** 1 Programa de Pós-Graduação em Ecologia, Instituto de Biologia, Universidade Estadual de Campinas (Unicamp)13083-970, Campinas, São Paulo, Brazil; 2 Departamento de Biologia Vegetal, Instituto de Biologia, Universidade Estadual de Campinas (Unicamp)13083-970, Campinas, São Paulo, Brazil; 3 Departamento de Genética, Evolução e Bioagentes, Instituto de Biologia, Universidade Estadual de Campinas (Unicamp)13083-970, Campinas, São Paulo, Brazil

**Keywords:** Dioecy, floral display, reproductive phenology, reproductive strategy, resource allocation, sex ratio

## Abstract

Reproductive strategies, sexual selection, and their relationship with the phenotype of individuals are topics widely studied in animals, but this information is less abundant for plants. Variability in flowering phenology among individuals has direct impact on their fitness, but how reproductive phenology is affected by the size of the individuals needs further study. We quantified the flowering intensity, length, and reproductive synchronization of two sympatric dioecious Wild Nutmeg tree species (*Virola*, Myristicaceae) in the Brazilian Atlantic forest, and analyzed its relationships with tree size. Two distinct strategies in flowering timing and intensity were found between species (annual versus biennial flowering), and among individuals in the annual flowering species (extended versus peak flowering). Only for the annual flowering species the reproductive output is related to tree size and large trees present proportionally higher flower coverage, and lower synchronization than smaller ones. Flowering is massive and highly synchronized in the biennial species. Sex ratios are not different from 1:1 in the two species, and in the two segregated reproductive subgroups in the biennial flowering species. The biennial flowering at individual level is a novelty among reproductive patterns in plants, separating the population in two reproductive subgroups. A proportional increase in the reproductive output with size exists only for the annual flowering species. A biennial flowering can allow resource storage favouring massive flowering for all the individuals diluting their relationship with size.

## Introduction

Animal reproductive strategies have been widely studied for many decades for different taxonomic groups, and the implications of particular phenotypic characteristics (e.g., body mass) in the reproductive success of the individuals are understood for many species (Andersson 1994). Despite plant sexual systems have been widely studied, more information is needed about the particular reproductive strategies of the individuals. Regardless of previous discussion about the importance of phenology as a selective factor (Ollerton and Lack 1992; Fox and Kelly 1993), recent analyses support flowering phenology as an important component mediating the fitness of the individuals (Munguía-Rosas et al. 2011bb). Studies have shown that different flowering phenologies can act as adaptive reproductive strategies, but also that external factors can influence the evolution of reproductive phenologies (Kudo 2006). The focus has been on species or population mechanisms and despite variation in flowering phenology among individuals within a population has been well documented, few studies have analyzed the factors affecting it (Primack 1980; Dieringer 1991; Ollerton and Lack 1998). Among these factors, size of individual plants has been found to influence their flowering phenology (Schmitt 1983; Dieringer 1991; Ollerton and Lack 1998; Torimaru and Tomaru 2006; Munguía-Rosas et al. 2011a), and probably its reproductive performance as well, mediating the attraction of animal vectors dispersing the pollen (Klinkhamer et al. 1989). Plant size can influence the acquisition of resources, especially in forest ecosystems where light incidence decreases considerably from canopy to understory strata. Especially for tree species, the great variation in size during their reproductive life can be related to variation in flowering phenology as light availability increases, and growth rate decreases as the individual grows (Muller-Landau et al. 2006).

A general hypothesis establishing that tree size influences the reproductive output and the phenological patterns of the individuals (flowering intensity and timing) can be proposed, with the prediction that proportional reproductive output will increase as does the size of the individuals. However, many phenological strategies are present among plant species (Newstrom et al. 1994; Fenner 1998; Sakai 2001), probably affecting their energetic requirements and allocation of resources, but also the way the reproductive output of the individuals of each species changes as they grow. For example, in dioecious tree species individuals of different sex are expected to administrate energy differently during their reproductive life and during each reproductive cycle and the relationships between size and reproductive output are expected to differ between them (Armstrong and Irvine 1989; Thomas and LaFrankie 1993; Queenborough et al. 2007). Also in populations with biased sex ratios it is possible that reproductive output and probably reproductive phenology could be modified as a response to different pressures caused by interference of conspecifics (see Weiner 1988). For this reason, analyzing the sex ratio within populations is important for the interpretation of reproductive phenology. Despite the huge diversity of species, the difficulty to quantify the reproductive output for large plants, and especially for trees, make that these relationships are poorly understood for this group.

Here, we test for an increment in reproductive output as size increases for individuals of two sympatric dioecious species of Wild Nutmeg trees, *Virola* (Myristicaceae) with contrasting phenological strategies in the Brazilian Atlantic rain forest. Reproductive output is here considered as flower/fruit coverage, while phenology comprises the flowering timing and length affecting the reproductive synchrony within the population and the reproductive performance of the individuals. We also analyzed the sex ratio in the total populations and in different size classes in order to determine if biased sex ratios are present and can influence the phenological patters observed in these populations.

## Material and Methods

### Study species and study site

**Figure 1:**
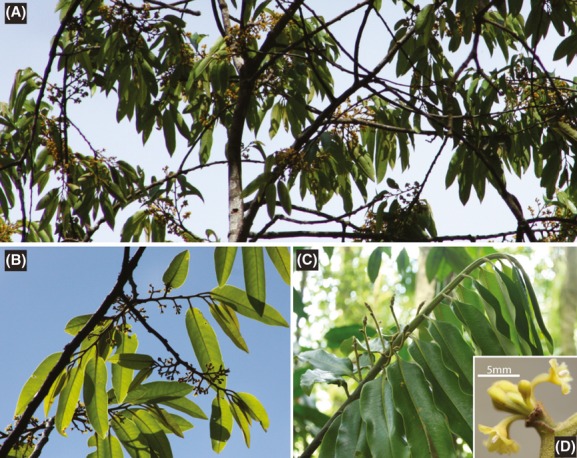
(A) Section of the crown of a male *Virola gardneri* with inflorescences in all terminal branches (coverage = 100%). (B) Detail of reproductive flowering twigs of *Virola gardneri*. (C) Terminal branch of *Virola bicuhyba* with developing inflorescences. (D) Male flowers of *Virola bicuhyba*.

*Virola bicuhyba* Schott ex Spreng. and *V. gardneri* (A. DC.) Warb. are dioecious endemic to Brazil and the only two species of the genus found in the Atlantic Rain Forest biome. They occur commonly in sympatry and produce inflorescences at the terminal parts of the branches. The flowers are small with three petals (rarely four), and 5 mm in diameter for both sexes (Fig. 1D). Flower color is light yellow and flower visitors are mainly flies of different groups (M. Fernández Otárola, personal observations). The study was conducted in the Serra do Mar State Park, Núcleo Picinguaba (47,500 ha), São Paulo, Brazil (23°22'S, 45°05'W). *Virola bicuhyba* and *V. gardneri* can reach up to 44 and 35 m, respectively, in the studied area. The sampled area is a continuous primary forest, classified as tropical moist evergreen according to Holdridge (1947). Average annual precipitation for the period 2008–2010 was 2918 mm, and average temperature varies between 18 and 27°C along the year (Ubatuba climatic station, Instituto Agronômico de Campinas).

### Phenological measurements and analyses

Within a permanent plot of 10 ha, 47 reproductive individuals of *V. bicuhyba* and 31 of *V. gardneri* were followed for 24 months. Phenological observations were made every month from May 2009 to April 2011. We estimated the proportion of the crown covered by inflorescences and fruits each month, and total flowering length. We also calculated the flowering synchronization and light incidence on each individual. As a direct account of inflorescences or fruits was not possible, we implemented a new technique that we called crown partition phenological accounting which divided each crown into small, equal size parts (the number will depend on crown size and structure and defined by the researcher) which are easier to sample. Each section of the crown is defined based on reference points (e.g., branches or irregularities), and observed with binoculars to calculate the proportion covered by reproductive structures. The coverage by flowers and fruits in each section is then estimated separately and finally the proportional values of all sections are summed to obtain the full crown coverage. Only the terminal part of the branches produce flowers in *Virola bicuhyba* and *V. gardneri* (Fig. 1), and the crown were divided into four equal size sections for coverage estimations in the two species. These values represent the standardized allocation to reproduction among individuals and not the quantification of reproductive structures. These monthly values were summed to obtain the total flower coverage, this is equivalent to calculate the area under the curve of flower production over time for each individual, and this value is interpreted as the number of crowns full of flowers produced during the full study period for each individual. This new method offers the advantage of being simple, cheap, easy to implement, and allow the comparison of phenological intensity among large amounts of individuals which is not possible with low-resolution methods currently used (e.g., Fournier).

Understory net traps are not feasible to be used for flower accounting in these species as flowers are fragile, small, and rapidly decompose. Also an estimation based in understory traps would require constant sampling not possible at the area which is a common limitation in phenological studies. Also, understory traps do not allow an adequate accounting of flowers, or fruits for comparisons among individuals, despite they are an effective method for community-based approaches. Especially for fruits, net traps can underestimate production; *Virola* species are an important food source for birds and other animals along all their distribution and few fruits are open every day and most of them are consumed (e.g., Howe and Kerckhove 1981). Also, as our intention was to compare flower and fruit production among individuals, we needed a standardized method to estimate total production of reproductive structures, and direct accounting of crown coverage is a direct and easy to implement technique to achieve this objective.

The first 6 months of observations were excluded to have a common period of data for all the individuals (19 months) because not all of them were followed since the first census. These 6 months are outside the high flowering season and their inclusion in the analyses does not change the results.

The flowering synchrony was evaluated with the Augspurger index (Augspurger 1983), which compares the overlap of the flowering time of each individual to the rest of the population (month was used as the unit for comparison). The index for three individuals is based on 1 year of observations; all others are based on the full study period.

### Illumination measurements

As tree size is related to the exposure in the canopy and the availability of resources, a crown illumination index – modified from Clark and Clark (1992) – was used to measure the light incidence on each tree. This index is widely used for the estimation of light incidence on tropical trees and is highly correlated with other more precise methods to evaluate light incidence (as hemispherical photographs) which demonstrate that this method is a good measure of the light interception achieved by large individual trees (see Keeling and Phillips 2007).

The scores were as follows: 1 (no direct lateral or overhead light), 1.5 (little direct lateral light, no overhead light), 2 (some direct lateral light, no overhead light), 2.5 (substantial direct lateral light, no overhead light), 3 (some overhead direct light), 4 (full overhead direct light; in canopy), 4.5 (full overhead direct light; substantial direct lateral light), and 5 (full overhead and lateral direct light; emergent). The value of 4.5 was included here as some trees in areas of high slope present this pattern of light incidence which is not considered by the index in their original form. An index value was given monthly during phenological observations; the average value for each tree was used in the analyses as an ordinal variable.

### Statistical analyses

Diameter at breast height (DBH) was used as a measure of tree size as it is highly correlated with the crown volume (*r* = 0.79 and *r* = 0.69 for *V. bicuhyba* and *V. gardneri*, respectively, *P* < 0.0001 for both species; M. Fernández Otárola, unpublished data). The relationships of total flower coverage, synchrony, and flowering length with sex and size were analyzed with ANCOVA using DBH as covariate. The Box-Cox transformation was used for the time of flowering, and total flower coverage (Sokal and Rohlf 1995). The flower coverage during the 2 months of flowering overlap between the species per year was compared with a nested ANOVA (year was nested in the species to control for the difference in the phenological pattern – annual versus biennial – of the individuals). We perform linear regressions to explore the relationship between tree size (DBH) and fruit coverage for the females of both species, and a simple correlation was used to determine the similarity in the flowering coverage among successive flowering episodes for the males in *V. bicuhyba*. Ordinal logistic models were used to analyze the relation between light index (average illumination index values) and total flower coverage per species and sex.

### Sex ratios

The sex of each tree was determined by the analysis of their flowers and presence of fruits during phenological observations. Sex ratios were compared using chi tests. To test sex ratios bias across different reproductive sizes, we categorized the reproductive individuals into three groups according to their DBH (small 15 - ≤ 30 cm, medium >30 - ≤50 cm, and large individuals >50 cm) in order to represent the size range in the population while keeping sufficient sample size. The sex ratio analyses include additional individuals not considered in the other analyses.

## Results

**Figure 2:**
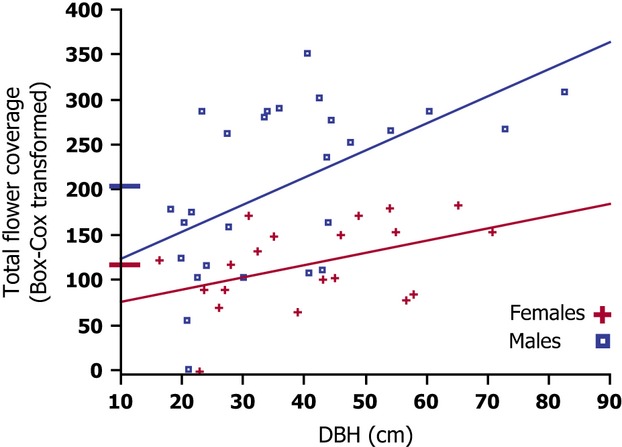
Relationship between total flower production and diameter at breast height according to sex for individuals of *Virola bicuhyba*. Thick lines on the dependent axis are the mean value for each sex according to each color.

**Figure 3:**
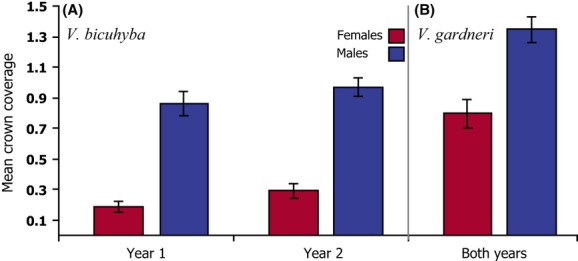
Average proportion of the crown area covered by flowers (± SE) during the first 2 months of the reproductive season (flowering overlap between the species) according to species and sex. (A) Annual data for *Virola bicuhyba*. (B) since *V. gardneri* is biennial each individual reproduces once during the study period and data for the 2 years were combined.

*Virola bicuhyba* showed an annual flowering pattern with the flowering season extending for up to 10 months a year for some individuals (Fig. S1A,B in Supporting Information). Large individuals of the two sexes exhibited higher proportional flower coverage than small individuals in this species (Table 1). Flower coverage was higher for males than for females in both species (Table 1 and Fig. 2), and higher in *V. gardneri* than in *V. bicuhyba* (Table 2, Fig. 3). In *V. bicuhyba*, the relationship of illumination and total flower coverage was not significant for both sexes (males: χ^2^ = 3.03, *P* = 0.08; females: χ^2^ = 0.86, *P* = 0.35); in this species similar crown coverage of each male between the 2 years suggests nonrandom differences in reproductive potential in the short time (*r* = 0.93, *P* < 0.0001, *n* = 24).

**Table 1:** ANCOVA comparing the flower coverage and its relation with tree sex and size (DBH) in *Virola* spp.

Factor	*Virola bicuhyba*			*Virola gardneri*		
	
	df	*F*	*P*	df	*F*	*P*
Sex	1, 42	29.71	<0.0001	1, 27	13.66	0.001
DBH	1, 42	14.3	0.0005	1, 27	0.14	0.71
Sex*DBH	1, 42	1.83	0.18	1, 27	0.92	0.34

**Table 2:** Nested ANOVA comparing the flower coverage during the 2 months of flowering overlap, between species, sexes, and years of study (data of both years were pooled for *Virola gardneri*)

Factor	df	*F*	*P*
Species	1	8.37	0.0048
Sex	1	19.29	<0.0001
Species*Sex	1	1.83	0.18
Year (Species)	1	1.43	0.23

**Figure 4:**
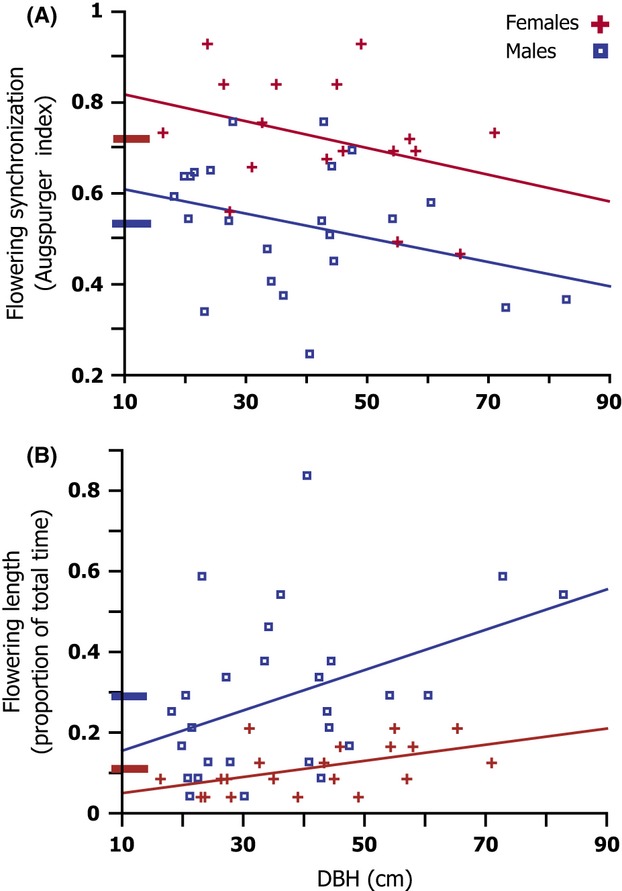
Relationship between diameter at breast height (DBH) and: (A) flowering synchronization; (B) flowering length, according to sex for individuals of *Virola bicuhyba*. Thick lines on the dependent axis are the mean value for each sex according to each color.

The biggest trees in *V. bicuhyba* were less synchronized in their flowering (*F*_1,42_ = 5.7, *P* = 0.02) and this pattern did not differ between the sexes (*F*_1,42_ = 0.03, *P* = 0.85; Fig. 4A), however, females were on average more synchronized than males (*F*_1,42_ = 21.3, *P* < 0.0001; Fig. 4A). Big trees flowered for longer periods than smaller individuals in *V. bicuhyba* (*F*_1,46_ = 10.3, *P* = 0.002) and flower production in males extended longer than females (*F*_1,46_ = 23, *P* < 0.0001; Fig. 4B). The relationship between flowering length and tree size did not differ between the sexes (*F*_1,46_ = 0.005, *P* = 0.94). Large females presented a proportionally higher fruit coverage than smaller ones (*r*^2^ = 0.3, *P* = 0.01).

Flower production in *V. gardneri* is highly synchronized and last for 2 months each year (Fig. S1C,D). While at the population level flowering individuals were observed annually, we detected that individuals (males and females) flower every other year, and we will refer to this as a biennial flowering pattern. This flowering pattern persisted for all the reproductive individuals over 5 years of observations (2008–2012). Among the reproductive trees, 53% flowered 1 year and the remaining 47% another year dividing the population into two isolated reproductive subgroups. *Virola gardneri* presents nonsignificant relationships between tree size and flower or fruit coverage for males and females (Table 1). In *V. gardneri*, the relationship of illumination and total flower coverage was not significant for both sexes (males: χ^2^ = 0.28, *P* = 0.6; females: χ^2^ = 0.09, *P* = 0.77). Average synchronization index values did not differ between males and females in this species (0.87 ± 0.13 and 0.92 ± 0.1, respectively; *t* = 1.44, df = 22, *P* = 0.16).

The two species showed identical sex ratios not different from 1:1 (54.5% males; *V. bicuhyba*: *n* = 55, χ^2^ = 0.45, df = 1, *P* = 0.5; *V. gardneri*: *n* = 33, χ^2^ = 0.27, df = 1, *P* = 0.6). No differences from a 1:1 sex ratio were found over DBH categories in both species (*V. bicuhyba* χ^2^ = 1.64, df = 2, *P* = 0.44; *V. gardneri* χ^2^ = 1.6, df = 2, *P* = 0.45). In the case of *V. gardneri*, unbiased sex ratios were found for both reproductive subgroups (even and odd years) within the population (χ^2^ = 0.06, df = 1, *P* = 0.8; χ^2^ = 0.25, df = 1, *P* = 0.62, respectively).

## Discussion

Selection on reproductive phenology can determine different reproductive strategies among species (Kudo 2006), but our data also indicate that variation among individuals can be important during ontogeny. We identified two main phenological patterns between the species – annual flowering versus biennial flowering –, but also variation between two synchrony extremes was exhibited by the individuals within a species and related to their size and sex: flowering only during the population peak (peak flowering), and extended flowering.

### Reproductive phenology of the individuals

Reproductive synchronization is an important trait in sexual organisms enhancing the chance of mating. Considering the variability in reproductive phenology among individuals, extended flowering individuals showed large flower coverage during the peak and kept flowering for several months. *Virola gardneri* males and females showed only peak flowering, whereas *V. bicuhyba* exhibits the full range of variation between the two extremes, the males being more extended than females (i.e., lower average values of the synchronization index). The presence of these strategies within a population will create skewed flowering distribution which has been related to competition for pollinators (see Thomson 1980). In *V. bicuhyba*, variation in the flowering phenology related to plant size reflects different reproductive allocation at different stages of the reproductive life. Light incidence alone does not explain crown coverage by flowers and fruits, and tree size is the factor with the larger relevance on reproductive effort. When tree size is about its maximum, vegetative growth slows down (Muller-Landau et al. 2006), and more resources can be allocated to reproduction producing proportionally higher flower coverage in males and fruit coverage in females. By this time, an extended flowering can increase the number of mates in the population.

In *V. bicuhyba*, highly synchronized males (peak flowering) can potentially fertilize the majority of females, as most of them flower during the peak. This strategy could be selectively favored for small individuals with more limited resources. Nevertheless, extended flowering individuals – mostly large individuals with abundant resources – flower intensely during the peak, but also out of it, and can potentially have reproductive access to the highly synchronous peak flowering females, as well as those few extending their flowering outside the peak population-wide flowering. This could give large males an advantage in extending their flowering, in other case, low synchronization should be counterselected. An extended strategy can potentially favor long-distance pollen dispersal, in cases where a gradient in flowering time occurs among neighboring populations as has been found for other species at variable spatial scales or over environmental gradients (Levin 2009; see Brito and Sazima 2012 for an example in the area here studied). In the case of females, an extended flowering does not show as many implications as it does for males because reproductive success will be determined by fruit production and different resource allocation and selective pressures will be present. Pollen is abundant during the flowering peak and few pollen grains could be required for fertilization, since just one ovule is present in each flower (but see Armstrong and Irvine 1989). This could explain the rarity of extended flowering in females, and the comparable short periods of flowering in relation to males.

### Reproductive phenology of the species

We report a novel reproductive phenological pattern in *V. gardneri* in which flowering occurs annually in the population, although it is composed of biennial flowering individuals, half of them flowering each year. This biennial reproductive phenology could allow resource storage and massive flowering episodes explaining the absence of any relationship between tree size and reproductive output. The entrance of newly recruited individual to any reproductive subgroup should keep the genetic cohesion in this population, as the onset of reproduction should be triggered by physiological conditions. In fact, initial reproductive activity is highly related to tree height for both species, and reproductive subgroups in *V. gardneri* are genetically indistinguishable (M. Fernández Otárola, unpublished results).

Reproductive interference among species in the community creates a strong selective factor on the way reproductive characters as flowering phenology evolve (Pfennig and Pfennig 2009). The biennial reproduction of *V. gardneri* can be related to this and can contribute to coexistence with other species via stabilizing mechanisms that increase intraspecific relative to interspecific competition (see Chesson 2000; Lankau 2011). The extended strategy described for *V. bicuhyba* may also fit this view of intraspecific competition, as those trees flowering for a long time can potentially produce more offspring. Similar results were found by Lack (1982) while studying a pair of sympatric, self-incompatible *Centaurea* species (one species extended their flowering after a period of reproductive overlap with their congener), and support the idea that pollinators can be a limiting resource when similar, related species live in sympatry.

### Sex ratios

Identical unbiased sex ratios were found in the populations of the two species. The sex ratio in *V. bicuhyba* and *V. gardneri* is also unbiased when partitioned in size categories indicating that sex expression related to size and differential mortality rates play no role in this parameter. Cases of both, biased and unbiased sex ratios in dioecious plants are common in the literature (Opler and Bawa 1978; Field et al. 2012; Sinclair et al. 2012). In fact, sex ratios have been found to be related to growth form, and pollination and dispersal ecology (Field et al. 2012; Sinclair et al. 2012). Most species of Myristicaceae analyzed including various *Virola* species showed unbiased sex ratios (Ackerly et al. 1990; Queenborough et al. 2007). Sex in Myristicaceae seems to be genetically determined, but the exact mechanism of sex determination is still unknown. Under this scenario, biased sex ratios are the response to long-term ecological/evolutionary processes, and not the effect of environmental variability. Equal cost to produce each sex and the absence of large differences in their specific environmental requirements can be responsible for the similar sex ratios found here.

### Other considerations

Both species presented great variability in flower coverage, but for the males of *V. bicuhyba*, similar crown coverage of each individual between the 2 years suggests no differences in reproductive potential in the short time. Despite this factor, individuals with similar sizes can present great variation in the crown coverage and time of flowering, showing that these characteristics are not fully dependent on tree size. In fact, previous studies have demonstrated the heritability of traits related to flowering phenology for a diverse group of plants (Elzinga et al. 2007). Variability in flowering intensity can theoretically allow the differential attraction of pollinators and the occurrence of sexual selection in plant species (Willson 1994; Skogsmyr and Lankinen 2002), but these factors require further study and no information is available for this species or other in the genus to our knowledge. It is unknown how environmental factor influence the flowering output in these species and how variability among reproductive episodes is distributed among individuals.

## Conclusions

Sympatric congeneric species can markedly differ in their reproductive phenology as this is directly related to their reproductive strategy. Despite an average phenological strategy at the species level, individuals within a population can differ in their reproductive phenology. This variability can be related to the ontogeny, and large individuals can present larger flowering intensity and flowering time which reduces their overall synchrony with the rest of the population. This effect differs between the sexes, the males showing higher variability than females which is related to the optimal reproductive allocation in reproductive structures (flowers or fruits) between them. Finally, the relationship between size and phenology is species specific and is dependent on the general species phenological strategy, being absent in one of the species here studied.

## Supporting Information

**Figure S1.** Flower coverage per individual over the study period for *Virola bicuhyba* and *Virola gardneri*.
