# Supplementary material for: Tree size and its relationship with flowering phenology and reproductive output in Wild Nutmeg trees
Source: Ecol Evol. 2013 Aug 29;3(10):3536–44. doi: 10.1002/ece3.742 (PMC3797497; doi:10.1002/ece3.742)
Supplement: Supplementary file 1 [file ece30003-3536-SD1.pdf]

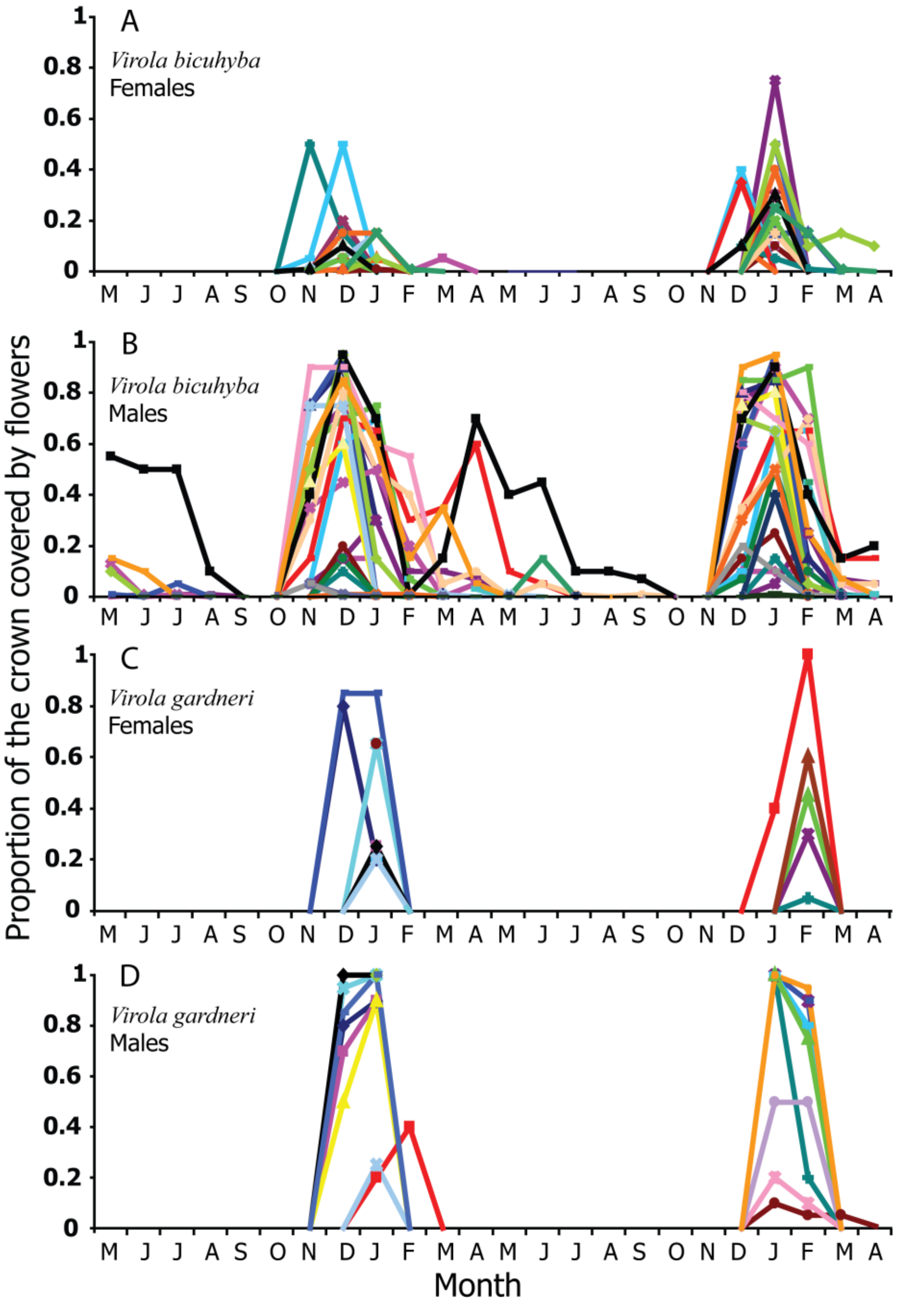

Fig. S1. Flowering curves showing the monthly proportion of the tree crown covered by flowers according to species and sex. Each line/color represents an individual.
